# Epidemiology of Trichomoniasis in South Korea and Increasing Trend in Incidence, Health Insurance Review and Assessment 2009-2014

**DOI:** 10.1371/journal.pone.0167938

**Published:** 2016-12-09

**Authors:** So-Young Joo, Youn-Kyoung Goo, Jae-Sook Ryu, Sang-Eun Lee, Won Kee Lee, Dong-Il Chung, Yeonchul Hong

**Affiliations:** 1 Department of Parasitology and Tropical Medicine, Kyungpook National University School of Medicine, Daegu, Republic of Korea; 2 Department of Environmental Biology and Medical Parasitology, Hanyang University College of Medicine, Seoul, Republic of Korea; 3 Division of Malaria and Parasitic Diseases, Korea National Institute of Health, Korea Centers for Diseases Control and Prevention, Osong, Republic of Korea; 4 Medical Research Collaboration Center in KNUH, Kyungpook National University School of Medicine, Daegu, Republic of Korea; Fudan University, CHINA

## Abstract

Trichomoniasis, which is caused by *Trichomonas vaginalis*, is one of the most common non-viral sexually transmitted infections; however, limited population-based data are available that describe patterns and trends of the disease. We summarized insurance claims of trichomoniasis cases reported during 2009–2014 to South Korea Health Insurance Review and Assessment Service. The average annual incidence in South Korea was 276.8 persons per 100,000 population, and a substantial sex-associated variation was observed. The incidence rate among female subjects trended upward over 6 years, that is, it increased from 501 in 2009 to 625.8 in 2014 per 100,000 female population, which indicates a 25% overall increase. This trend was sharpest in the ≥60 years group of female population. However, a 66% decrease in incidence rates was observed among male subjects (23.7 in 2009 to 15.7 in 2014 per 100,000 male population). Further, substantial decrease was observed in the ≥40 years groups of male population. The incidence of trichomoniasis varied across regions and was the highest in Jeju province of South Korea. Overall, as the incidence of trichomoniasis appears to have increased in South Korea during 2009–2014, the disease burden is increasing; hence, there is a need to better understand the disease transmission.

## Introduction

Trichomoniasis caused by the *Trichomonas vaginalis* (*T*. *vaginalis*) manifests vaginitis and cervicitis in women, and serious complications including infertility, preterm birth, and pelvic inflammatory disease potentially occur [1–8]. Although incidence of trichomoniasis in men was relatively lower compared with that in women, *T*. *vaginalis* infection can cause nongonococcal urethritis and might result in prostatitis, epididymitis, and infertility in men [9–11]. However, majority of men or women with *T*. *vaginalis* infection are asymptomatic and represent a risk of transmission to their sexual partners as a reservoir of *T*. *vaginalis* [2, 12–14]. Trichomoniasis is one of the most common non-viral sexually transmitted diseases (STDs). Although about 248 million new cases of trichomoniasis have been reported annually worldwide [12, 15], the true incidence has not been well established. According to previous studies, the incidence and prevalence of trichomoniasis varies by the surveillance population, time, and region; however, high incidence rate is generally presented in women and older groups compared to men or younger groups, which is inconsistent with other STDs, such as chlamydia or gonorrhea [16]. In the United States, the prevalence of *T*. *vaginalis* infection is estimated to be 3.1% among women aged 14–49 years [17], and 2.3% among adolescents [18] based on a nationally representative study. Population-based studies in Asia show lower rates, that is, 2.9% in Shandong Province in China [19] and 1% in rural distinct of Vietnam [20]. Until now, various prevalence levels of trichomoniasis have been reported in South Korea depending on the researchers and study populations [21]. However, population-based data about the incidence and trends of trichomoniasis in South Korea are limited. It is imperative for men and women to monitor changes associated with trichomoniasis in order to develop appropriate preventive measures and control programs for reducing the disease burden. This study determined the incidence of trichomoniasis in South Korea and examined the incidence rates of trichomoniasis with age-, sex-, and region-specific differences from 2009–2014 using clinical data that were obtained from the national health insurance database containing the claims-related data for all the healthcare facilities in South Korea.

## Materials and Methods

### Study design

The data that were used in this analysis were obtained from the South Korea Health Insurance Review and Assessment Service (HIRA), which contains a series of health insurance population-based longitudinal databases that encompass approximately 97% of the people living in South Korea (https://www.hira.or.kr/eng/) [22] (S1 Table). Analysis in this study was descriptive and restricted to the evaluation of demographic variables of the registered health insurance population in the HIRA. All residents of South Korean have a 13-digit number used for registration of individual patients in the medical insurance system, which has been used for nationwide epidemiological surveys. In this study, to remove overlapping medical records of patients who received multiple episodes of treatment for the same disease at the same or different locations, or for those who transferred to other hospitals or clinics for further treatment, data were collected using the registration number to represent all treatment for one patient in one year. The HIRA collects the records of all the patients at hospitals or clinics, which include diagnoses, procedures, prescription records, demographic information, and direct medical costs. It includes 99% of hospitals and clinics under mandatory single-payer health insurance system [23]. Studies have systematically validated the accuracy and reliability of the HIRA data [24]. We obtained the extracted insurance claims for STDs including trichomoniasis with demographic information from 2009 to 2014 from the South Korea HIRA database using code A59 (A59.0, A59.8, and A59.9) for trichomoniasis, A50–A53 for syphilis, A54 for gonorrhea, A55 and A56 for chlamydia, A60 genital herpes, which corresponds to the International Classification of Diseases ninth Revision codes for trichomoniasis diagnoses (131.00, 131.01, 131.02, 131.09, 131.8 and 131.9), venereal diseases due to *Chlamydia trachomatis* (099.5), gonorrhea (098), and syphilis (090–097), genital herpes (054.10) [25]. Incidence rates of trichomoniasis including other STDs were determined using the registered population in HIRA at the end of each year as the denominator, and it was defined as the number of patients per 100,000 persons registered in HIRA with health insurance. Both male and female patients were stratified into the following age groups: under 20, 20 to 29, 30 to 39, 40 to 49, 50 to 59, 60 to 69, and over 70 years. South Korea consists of eight cities, the capital of South Korea (Seoul), six metropolitan cities (Busan, Daegu, Incheon, Gwangju, Daejeon, and Ulsan) and one new administrative city (Sejong) and nine provinces (Gyeonggi, Gangwon, Chungbuk, Chungnam, Jeonnam, Jeonbuk, Gyeongbuk, Gyeongnam, and Jeju). Because Sejong was inaugurated in 2012 and its population is the smallest among metropolitan cities, the incidence rates of Sejong were excluded in regional analysis of incidence. Maps were created to evaluate nationwide differences in incidence rates, and changes in color intensity were used. Division of color shades was determined using six groups of incidence rates in a single map.

## Results

### Overall incidence and trends

During 2009–2014, trichomoniasis emerged as the most common STD among the total population of South Korea (average annual incidence rate = 276.8 patients per 100,000 persons) followed by genital herpes, chlamydia, syphilis, and gonorrhea (Table 1).

**Table 1:** Number of patients with STD and rate per 100,000 person-years in HIRA, South Korea from 2009 to 2014.

Years*	2009	2010	2011	2012	2013	2014	Average
**Syphilis**							
No. of patients	23,916	22,046	22,537	22,535	21,608	22,334	22,496
Incidence rate**	49.2	45.1	45.7	45.4	43.2	44.4	45.5
**Gonorrhea**							
No. of patients	21,451	19,704	18,924	16,962	14,191	14,139	17,562
Incidence rate	44.1	40.3	38.4	34.2	28.4	28.4	35.6
**Chlamydia**							
No. of patients	26,474	26,462	25,517	25,781	24,904	26,980	26,020
Incidence rate	54.5	54.1	51.8	51.9	49.8	53.6	52.6
**Trichomoniasis**							
No. of patients	126,572	120,771	125,703	123,875	156,262	160,543	137,036
Incidence rate	260.4	264.3	255.0	249.4	312.6	319.1	276.8
**Genital Herpes**							
No. of patients	113,549	118,820	123,858	122,525	117,555	119,776	119,347
Incidence rate	234	243	251	247	235	238	241

* For 2009–2014, data were reported for the calendar year ending December, 31.

** Number of patients per 100,000 persons registered in HIRA per year.

Among these STDs, annual incidence rates of trichomoniasis per 100,000 persons increased by 23%, from 260.4 in 2009 to 319.1 in 2014 (Table 2).

**Table 2:** Number of patients with trichomoniasis per 100,000 person-years in HIRA, South Korea from 2009 to 2014.

Age Group (in years)	2009			2010			2011			2012			2013			2014			Average*		
	Total	Male	Female	Total	Male	Female	Total	Male	Female	Total	Male	Female	Total	Male	Female	Total	Male	Female	Total	Male	Female
**Total**	260.4	23.7	501.0	264.3	22.5	510.0	255.0	19.6	493.9	249.4	20.0	481.8	312.6	17.7	611.0	319.1	15.7	625.8	276.8	19.9	537.2
**<20**	20.2	0.5	41.9	22.2	0.5	46.0	22.0	0.5	45.7	23.0	0.7	47.3	27.5	0.6	56.7	27.9	0.7	57.4	23.8	0.6	49.2
**20–29**	367.3	13.9	747.6	376.2	14.4	768.4	379.9	12.3	780.7	377.9	14.4	777.3	479.0	11.7	997.2	477.9	12.0	999.8	409.7	13.1	845.2
**30–39**	414.4	30.2	820.7	423.0	27.9	840.8	415.0	24.8	827.4	402.6	27.5	796.9	528.1	25.8	1,058.4	558.9	25.2	1,123.8	457.0	26.9	911.3
**40–49**	474.8	51.4	916.5	472.2	46.7	917.5	434.8	38.5	850.6	414.3	36.2	809.8	501.1	32.1	989.2	507.3	27.6	1,006.6	467.4	38.7	915.0
**50–59**	286.1	44.1	528.8	297.3	41.9	552.6	286.4	36.2	536.3	285.1	35.8	534.9	348.0	29.9	667.6	349.3	25.2	674.7	308.7	35.5	582.5
**60–69**	93.7	13.5	167.7	97.6	14.8	174.5	95.5	14.3	171.3	106.4	13.8	193.2	135.1	12.6	250.6	140.6	8.8	265.7	111.5	13.0	203.8
**70≤**	30.8	5.0	47.2	32.6	4.3	50.7	33.5	3.8	52.7	37.7	4.5	59.5	51.3	3.9	82.8	56.6	2.6	92.7	40.4	4.0	64.3

* Average incidence rate from 2009 to 2014.

**Fig 1:**
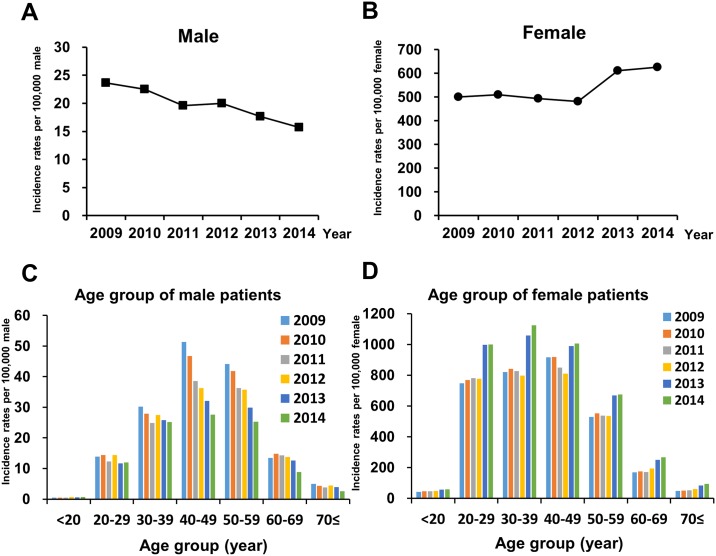
Incidence rate of trichomoniasis per 100,000 persons in HIRA by sex and age groups, South Korea 2009–2014. Annual incidence rate of trichomoniasis in male (A) or female (B) from 2009 to 2014. Sex-specific incidence rates of trichomoniasis according to each age group in male (C) and female (D) from 2009 to 2014.

Female individuals in South Korea had significantly high average annual incidence (537.2 patients per 100,000 female persons) than male individuals (19.9 patients per 100,000 male persons). The highest average incidence rate in female individuals was observed in the age group of 40 to 49 years (915.0 per 100,000 female), and this was followed by 30–39 years age group (911.3 per 100,000 female), which showed that the majority of trichomoniasis cases in female individuals was observed in those under 50 years of age (79% of total female patients). In female individuals, the incidence rates of trichomoniasis increased by 25%, that is, 501.0 in 2009 to 625.8 in 2014 per 100,000 female population (Table 2) (Fig 1B). There is substantial increase of incidence rate in the age groups ≥60 years, such as ≥70 (97%) and 60–69 (58%) from 2009 to 2014 (Fig 1D).

In male individuals, average incidence rates of trichomoniasis were the highest in the age group of 40–49 years (38.7 per 100,000 male), which was followed by 50–59 years age group (35.5 per 100,000 male) (Table 2); this shows that the majority of trichomoniasis cases were observed in male individuals who were aged >40 years (67% of total male patients). However, the incidence rates in male patients decreased by 66%, that is, 23.7 in 2009 to 15.7 in 2014 per 100,000 male population (Table 2) (Fig 1A and 1C). Notably, there is a substantial decrease in incidence rate in male in the age groups of ≥70, 40–49 and 50–59 years, that is, 48%, 46% and 43% from 2009 to 2014, respectively (Fig 1C).

### Regional variations

**Fig 2:**
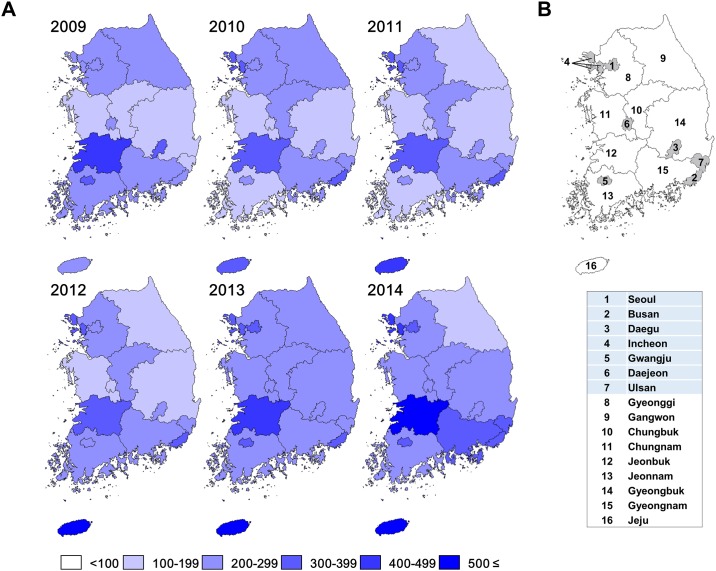
Nationwide trichomoniasis incidence rates according to administrative districts of South Korea from 2009 to 2014. (A) Incidence rates per 100,000 persons in HIRA were ranked into quintiles representation. (B) Administrative districts of South Korea. The numbers shown in the map (upper panel) corresponds to the 16 administrate districts (lower panel), 7 major cities (shaded) and 9 provinces in South Korea.

Subsequently, we examined the regional incidence rates of trichomoniasis stratified into six groups for administrative districts in South Korea from 2009 to 2014. As shown in Fig 2, the epidemiology of the incidence of trichomoniasis in South Korea showed the high level of regional variation.

Between 2009 and 2014, the incidence of trichomoniasis increased substantially in almost all regions in South Korea except Gangwon. There was a notable increase in the south regions, such as Jeju and Gyeongnam and in west regions of South Korea such as Jeonbuk, Chungnam and Incheon. Among South Korea’s provinces, Jeju had the highest average incidence rate of trichomoniasis (443.2 per 100,000 persons) followed by Jeonbuk (417.2 per 100,000 persons) during the study period (Table 3).

**Table 3:** The regional incidence rates of trichomoniasis cases per 100,000 persons in HIRA, South Korea from 2009 to 2014. Unshaded and shaded rows represent provinces and cities in administrative districts of South Korea, respectively.

City/Province	2009			2010			2011			2012			2013			2014			Average*		
	Total	Male	Female	Total	Male	Female	Total	Male	Female	Total	Male	Female	Total	Male	Female	Total	Male	Female	Total	Male	Female
**1. Seoul**	256.8	16.6	493.8	273.2	17.4	525.9	284.7	14.4	551.0	277.6	20.2	530.2	351.8	16.7	679.4	337.6	15.8	651.1	297.0	16.8	571.9
**2. Busan**	289.3	21.3	559.5	309.5	21.1	598.0	302.9	21.9	583.6	304.7	20.0	588.0	338.1	16.8	657.1	383.6	16.7	746.9	321.4	19.6	622.2
**3. Daegu**	354.6	25.4	691.2	273.6	21.0	529.4	245.5	19.4	473.8	219.2	17.5	422.3	262.2	11.3	514.4	263.7	8.7	519.7	269.8	17.2	525.1
**4. Incheon**	242.5	24.5	466.1	330.4	24.1	645.6	347.9	20.7	683.7	335.7	21.4	657.2	394.1	17.8	778.5	436.0	16.1	864.6	347.7	20.8	682.6
**5. Gwangju**	313.2	46.9	580.4	288.3	42.9	533.5	268.5	22.8	513.5	250.4	17.9	482.1	365.2	20.2	708.9	387.7	15.0	758.6	312.2	27.6	596.2
**6. Daejeon**	201.7	16.8	388.1	180.5	21.6	341.2	149.5	16.6	283.7	125.8	14.0	238.4	228.8	13.5	445.5	206.3	12.5	401.1	182.1	15.8	349.7
**7. Ulsan**	195.1	29.3	367.4	202.6	30.1	386.5	236.0	21.5	465.2	285.4	23.0	565.9	272.2	17.7	544.7	305.8	12.3	620.6	249.5	22.3	491.7
**8. Gyeonggi**	274.8	20.0	534.5	281.4	18.7	551.7	244.7	16.5	479.1	221.1	17.1	430.3	297.4	16.2	585.6	294.5	13.6	582.6	269.0	17.0	527.3
**9. Gangwon**	220.8	17.1	430.8	220.3	15.6	430.1	194.1	16.8	375.6	191.0	14.8	371.2	222.1	13.0	436.3	188.5	12.5	368.7	206.1	15.0	402.1
**10. Chungbuk**	184.4	20.1	355.0	281.7	20.2	551.5	280.0	22.1	545.8	238.7	16.4	467.5	271.6	13.0	538.2	277.3	10.3	552.7	255.6	17.0	501.8
**11. Chungnam**	188.3	18.5	365.4	159.9	22.3	302.9	157.4	15.9	304.7	186.1	13.7	365.3	287.9	11.2	576.3	276.6	12.1	552.9	209.3	15.6	411.3
**12. Jeonbuk**	426.6	22.8	841.8	366.7	19.9	717.5	346.4	15.7	680.5	371.3	16.4	729.0	476.5	15.3	940.6	515.4	12.2	1,021.1	417.2	17.0	821.7
**13. Jeonnam**	254.6	47.1	469.9	195.6	41.9	351.6	187.1	40.2	336.2	201.3	32.4	372.2	211.1	26.3	397.6	204.6	21.3	389.7	209.1	34.9	386.2
**14. Gyeongbuk**	185.3	33.3	343.8	188.9	31.5	350.6	182.4	28.5	340.6	168.1	26.3	313.6	219.0	19.4	423.8	275.5	17.9	539.8	203.2	26.1	385.4
**15. Gyeongnam**	229.8	39.8	427.0	219.2	30.3	414.4	224.7	28.6	427.5	259.3	31.7	494.1	295.4	30.3	569.1	320.6	24.8	626.8	258.2	30.9	493.2
**16. Jeju**	268.6	21.7	520.2	345.7	22.3	673.3	406.3	17.7	800.2	505.7	19.5	998.9	591.1	50.2	1,140.4	541.8	63.0	1,028.7	443.2	32.4	860.3
**Cities (1–7)**	264.7	25.8	506.6	265.4	25.4	508.6	262.2	19.6	507.8	257.0	19.1	497.7	316.0	16.3	618.3	331.5	13.9	651.8	282.8	20.0	548.5
**Provinces (8–16)**	248.1	26.7	476.5	251.0	24.7	482.6	247.0	22.4	476.7	260.3	20.9	504.7	319.1	21.6	623.1	321.6	20.9	629.2	274.5	22.9	532.1
**Total**	260.4	23.7	501.0	264.3	22.5	510.0	255.0	19.6	493.9	249.4	20.0	481.8	312.6	17.7	611.0	319.1	15.7	625.8	276.8	19.9	537.2

* Average incidence rate from 2009 to 2014.

**Fig 3:**
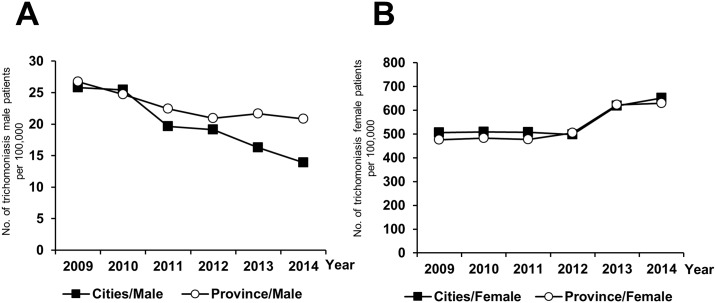
Sex-specific incidence rates of trichomoniasis in HIRA according to cities or provinces group of South Korea from 2009 to 2014 (A, male and B, female).

Although Jeju and Jeonbuk, as provinces in South Korea, have relatively higher incidence rates of trichomoniasis, the average incidence rate of trichomoniasis tends to be slightly higher in the cities (282.8 per 100,000 persons) than provinces (274.5 per 100,000 persons) (Table 3). From cities/provinces and male/female perspective, the incidence rates of male inhabitants in cities continuously decreased by 46%, that is, 25.8 in 2009 to 13.9 in 2014 per 100,000 male cities persons, and the decrease was relatively higher than that of male inhabitants in provinces (22%) (Fig 3A). However, the incidence rate of female patients showed similar increased trends both in cities (29% increase) and provinces (32% increase) during study period (Fig 3B).

## Discussion

From 2009 to 2014 in South Korea, trichomoniasis is the most common STD, and its incidence is increasing nationwide. In the United States, an estimated 7.4 million new *T*. *vaginalis* infections occur annually, which is considered to be approximately double the number of chlamydia and gonorrhea infections [26, 27]. In South Korea, annual incidence of trichomoniasis was approximately 3.1 times higher than that of chlamydia and gonorrhea combined (Table 1). As described in detail previously [28], microscopic examinations of vaginal wet preparations (or “wet mount”) are still routinely performed as a diagnostic test for trichomoniasis in most of the primary clinics and many hospitals in South Korea rather than the more sensitive nucleic acid amplification tests. The sensitivity of wet mounts for detecting *T*. *vaginalis* is only 50–70% [29–31]. Considering these situations, real incidence rates of trichomoniasis in South Korea could be higher than those observed in this study.

The global estimated prevalence of trichomoniasis is more common in female (8 times higher than male) [32]. From this study, it was determined that the incidence rate of trichomoniasis in female was 27 times higher than that in male population, indicating that the incidence of trichomoniasis in female in South Korea was extremely higher than that in male population or the incidence of trichomoniasis in male in South Korea was significantly lower than that in female population. *T*. *vaginalis* infection in male patients is hardly detected using diagnostic tests for female patients because of the low number of parasites in male urethral specimens that results in very low sensitivity. Until now, trichomoniasis has not been routinely considered in male patients attending urology clinics or hospitals in many countries including South Korea. Thus, it is likely that low incidence of trichomoniasis in male population may be caused by the absence of licensed diagnostic tests for male patients.

Based on this study, incidence of trichomoniasis in South Korea has increased, especially in 2013 and 2014 (Table 2). However, the incidence rate of trichomoniasis in male population gradually declined throughout the study period (Fig 1A). Thus, the increased incidence of trichomoniasis was due to the significantly increasing number of female patients. Interestingly, the incidence rate of trichomoniasis in female population was not changed or slightly decreased between 2009 and 2012 (from 501 to 482 per 100,000 female persons). However, a substantial increase was observed after 2013 (610 per 100,000 female persons) (Fig 1B). From 2009–2012, the incidence of trichomoniasis had been highest in the aged group 40–49 years in both male and female population. However, in male population above 40 years of age, especially 40–49 and 50–59 years, significant decrease of incidence was observed and was persistent throughout the study period. Whereas, in female age groups, especially 20–39 years, significant increase of incidence was observed after 2013. There is no clear single explanation for why incidence rate of trichomoniasis in male population decreased whereas that in female patients increased after 2012. It has been 11 years since the Special Law on Prostitution, prohibiting the buying and selling of sex and brothels, was implemented by the government of South Korea. Although those laws may affect the decreased incidence in male population, the increased incidence in female population cannot be explained. Although recently increased incidence of trichomoniasis in female is likely to be partially attributable to the introduction of more sensitive diagnostic tests; however, this also does not explain the decreased rates of male population through the study period.

It has been considered that *T*. *vaginalis* infection rates increase with age in contrast to other non-viral sexually transmitted infections [33]. The prevalence of trichomoniasis in women was the highest in the 51–60 years group, which was followed by the >60 years and 41–50 years groups in metropolitan areas with high STIs [34]. As shown in Fig 1D, the average annual incidence rate of trichomoniasis in female subjects gradually increased with age. However, this increase in incidence rate was not observed in women in South Korea aged >50 years. The average incidence rate in female subjects peaked in the age group of 40–49 years (915.0 per 100,000 female persons) and was followed by those in the age groups of 30–39 years (911.3 per 100,000 female persons) and 20–29 years (845.2 per 100,000 female persons) (Table 2). This indicates that the incidence rate of trichomoniasis among female subjects increased with age and a majority of the incidence of trichomoniasis was observed in female subjects 20–49 years. In contrast to trichomoniasis in female subjects, the association between *T*. *vaginalis* infection and older age in male subjects is not fully understood [35, 36]. Previous surveillances in the United States indicated that the highest prevalence of *T*. *vaginalis* infection was significantly associated with age (peaking at between 51 and 60 years of age) [37]. However, Wendel *et al*. reported that the prevalence of *T*. *vaginalis* infection in men aged ≤28 years was similar to that in men aged >28 years [38]. In this study, incidence rate of trichomoniasis in those aged <30 years was relatively lower compared with that of the other age groups of male subjects and corresponding age groups of female subjects. Thus, further studies are required to check whether trichomoniasis in male subjects <30 years is indeed rare or asymptomatic. During the revision of this manuscript, we received and analyzed the 2015 HIRA data for trichomoniasis (S2 Table). In 2015, the incidence rate of trichomoniasis in male individuals increased from 15.7 in 2014 to 16.5 (S1A Fig). According to age groups in the male population in 2015, a decreased trend of incidence of trichomoniasis was observed among those aged 40–49 and 50–59. However, there was a substantial increase among male individuals aged 20–29 (29%), 60–69 (33%), and ≥70 (42%) (S1C Fig), compared with those in corresponding age groups in 2014. Notably, this increase of trichomoniasis in male individuals was obvious in city populations, i.e., 13.9 in 2014 and 16.5 per 100,000 male individuals in 2015 (S1E Fig). In contrast to the male population, the incidence rate in the female population decreased by 9%, i.e., 625.8 in 2014 and 569.0 per 100,000 female individuals in 2015 (S1B Fig), mainly among those aged ≤20 (15%), 20–29 (14%), and 40–49 (9%) which groups were represented the majority of trichomoniasis cases in female individuals from 2009 to 2014 (S1D Fig). In contrast to male population in 2015, a decreased incidence rate was observed in the provincial female population (S1F Fig).

As shown in Fig 2, an increased incidence of trichomoniasis was mainly focused in south and west regions of South Korea. The highest incidence of trichomoniasis was observed in Jeju, which is a province in South Korea located at the southmost island. Although the highest increase in the average incidence rate of trichomoniasis was observed in Jeju and Jeonbuk, the trends of incidence in male or female population with trichomoniasis were different. With respect to the female, Jeju had the highest average incidence rate of trichomoniasis (860.3 per 100,000 female persons) followed by Jeonbuk (821.7 per 100,000 female persons). With respect to the male, Jeonbuk had the highest average incidence rate (34.9 per 100,000 male persons) followed the Jeju (32.4 per 100,000 male persons) during the study period (Table 3). However, the incidence of trichomoniasis increased in both male and female population in Jeju, whereas it decreased in the male population in Jeonbuk. Moreover, significant increase of incidence was observed in male population in Jeju, especially aged 30–59 years, from 2013 (S2 Fig). It is unclear why high incidence of trichomoniasis persisted in this region. In Jeju, the hospital admission rate for pelvic inflammatory disease is 1.5 times higher than that of the nation average of South Korea (1185 versus 768 per 100,000 female persons in 2014) (data obtained from HIRA). Thus, surveillance of STIs including trichomoniasis based on highly sensitive testing and control programs should be implemented to prevent further increase in the transmission in these regions of South Korea.

## Supporting Information

S1 Fig Incidence rate of trichomoniasis per 100,000 persons in HIRA by sex, age group, and cities or provinces groups in South Korea 2009–2015. Annual incidence rate of trichomoniasis in males (A) and females (B) from 2009 to 2015. Sex-specific incidence rates of trichomoniasis by age group in males (C) and females (D) from 2009 to 2015. Sex-specific incidence rates of trichomoniasis according to cities or provinces groups in South Korea from 2009 to 2015 (E, male and F, female).
(PPT)

S2 Fig Sex-specific incidence rates of trichomoniasis in two provinces, Jeju and Jeonbuk from 2009 to 2014 (A, male and B, female). (PPT)

S1 Table Number of persons registered in HIRA with national health insurance and proportion of those among total population of South Korea 2009–2014. (DOC)

S2 Table Number of patients with trichomoniasis per 100,000 person-years in HIRA, South Korea 2015. (DOC)
