# Supplementary material for: Epidemiology of Trichomoniasis in South Korea and Increasing Trend in Incidence, Health Insurance Review and Assessment 2009-2014
Source: PLoS One. 2016 Dec 9;11(12):e0167938. doi: 10.1371/journal.pone.0167938 (PMC5148063; doi:10.1371/journal.pone.0167938)
Supplement: S1 Fig — Annual incidence rate of trichomoniasis in males (A) and females (B) from 2009 to 2015. Sex-specific incidence rates of trichomoniasis by age group in males (C) and females (D) from 2009 to 2015. Sex-specific incidence rates of trichomoniasis according to cities or provinces groups in South Korea from 2009 to 2015 (E, male and F, female). (PPT) [file pone.0167938.s001.ppt]

## Slide 1
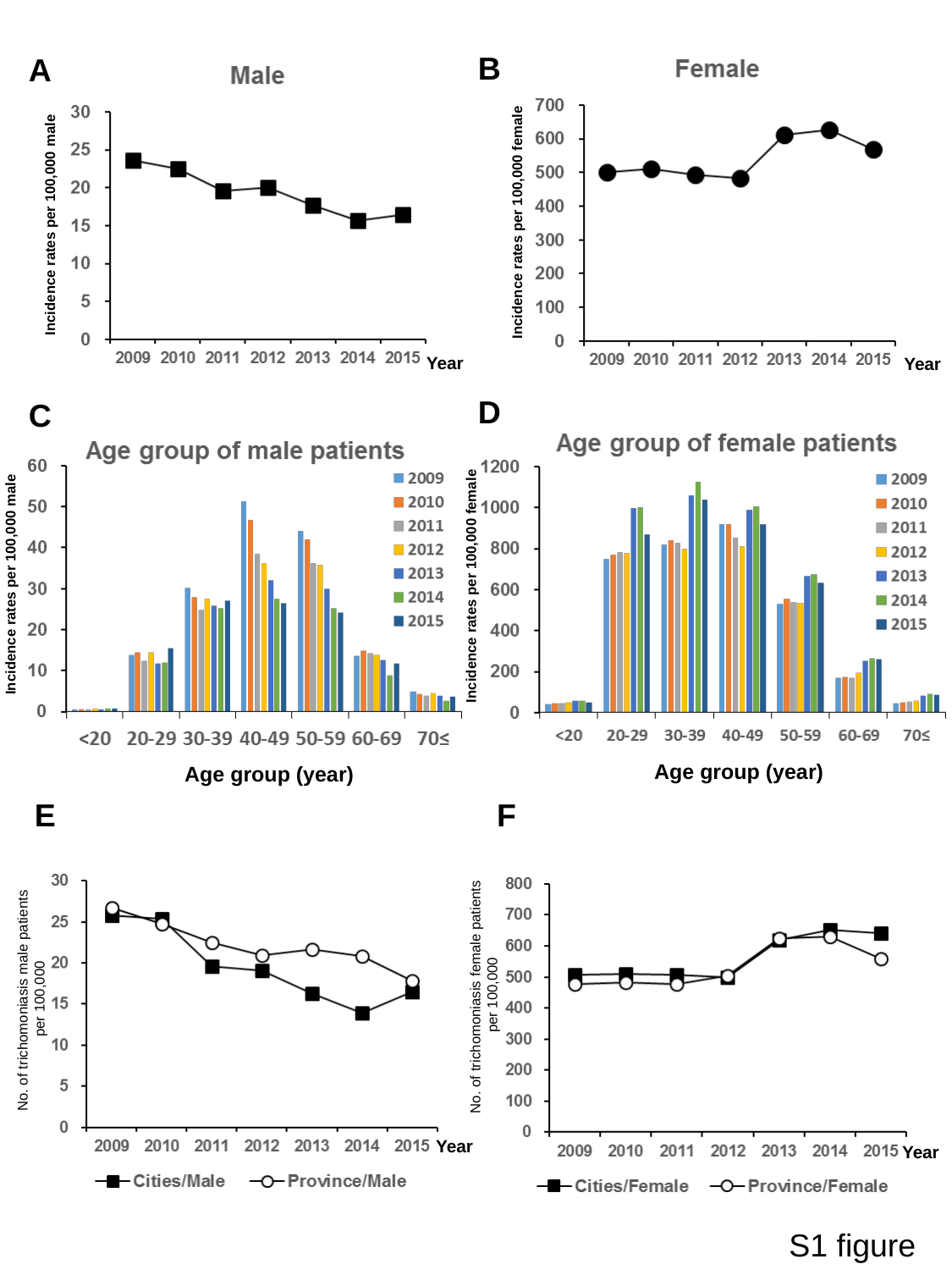

B
A
Incidence rates per 100,000 female
Incidence rates per 100,000 male
Year
Year
D
C
Incidence rates per 100,000 male
Incidence rates per 100,000 female
Age group (year)
Age group (year)
E
F
No. of trichomoniasis female patients
per 100,000
No. of trichomoniasis male patients
per 100,000
Year
Year
S1 figure
