# Supplementary material for: Epidemiology of Trichomoniasis in South Korea and Increasing Trend in Incidence, Health Insurance Review and Assessment 2009-2014
Source: PLoS One. 2016 Dec 9;11(12):e0167938. doi: 10.1371/journal.pone.0167938 (PMC5148063; doi:10.1371/journal.pone.0167938)
Supplement: S2 Fig — (PPT) [file pone.0167938.s002.ppt]

## Slide 1
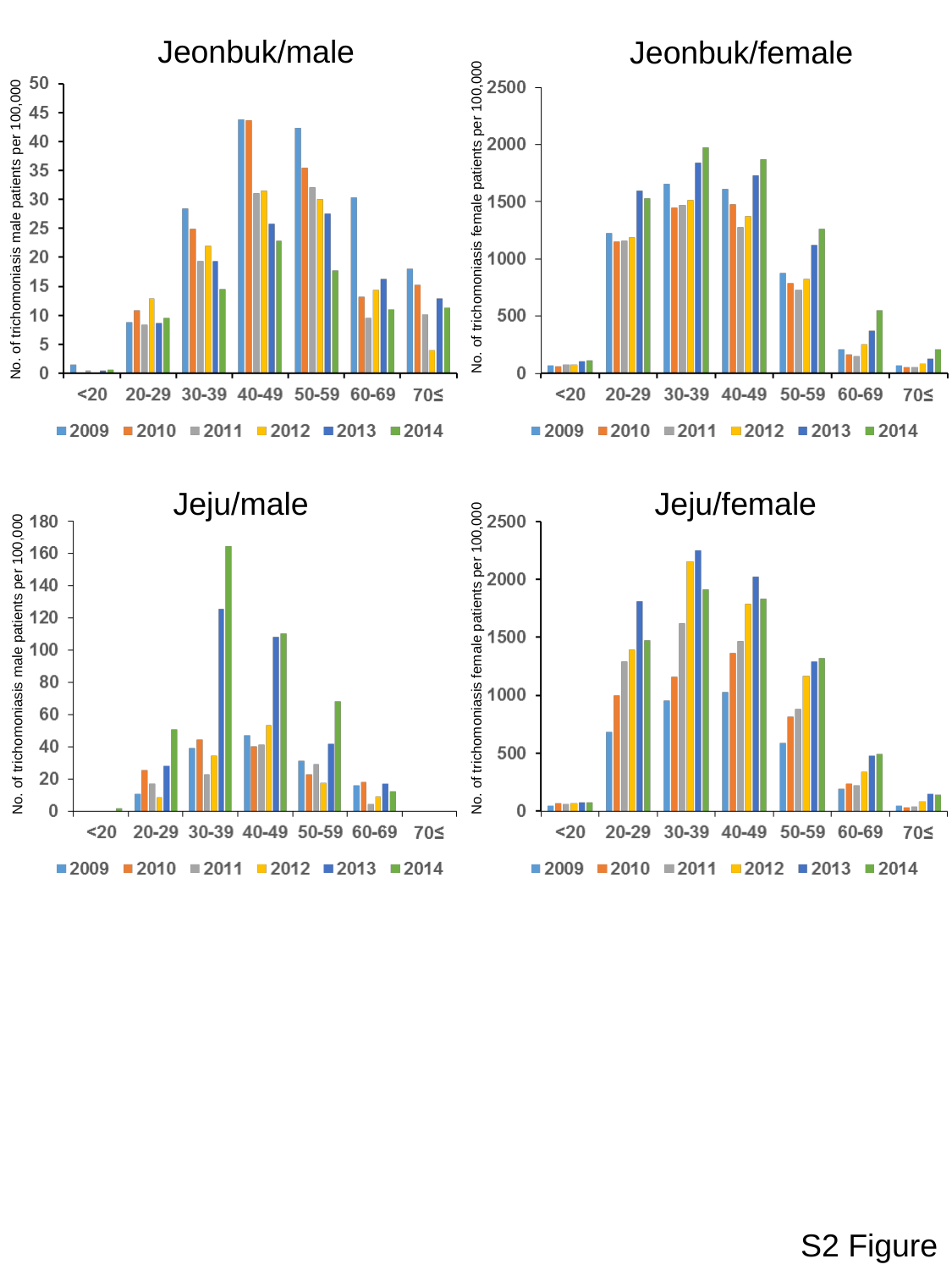

Jeonbuk/male
Jeonbuk/female
No. of trichomoniasis female patients per 100,000
No. of trichomoniasis male patients per 100,000
Jeju/male
Jeju/female
No. of trichomoniasis female patients per 100,000
No. of trichomoniasis male patients per 100,000
S2 Figure
