# Supplementary material for: Epidemiology of Trichomoniasis in South Korea and Increasing Trend in Incidence, Health Insurance Review and Assessment 2009-2014
Source: PLoS One. 2016 Dec 9;11(12):e0167938. doi: 10.1371/journal.pone.0167938 (PMC5148063; doi:10.1371/journal.pone.0167938)
Supplement: S1 Table — (DOC) [file pone.0167938.s003.doc]

**S1. Table. Number of persons registered in HIRA with national health insurance and proportion of those among total population of South Korea 2009-2014**

| **Years** | **2009** | **2010** | **2011** | **2012** | **2013** | **2014** |
| --- | --- | --- | --- | --- | --- | --- |
| **No. of health insurance** | 48,613,534 | 48,906,795 | 49,299,165 | 49,662,097 | 49,989,620 | 50,316,384 |
| **No. of total population** | 49,773,145 | 50,515,666 | 50,734,284 | 50,948,272 | 51,141,463 | 51,327,916 |
| **Proportion (%)** | 97.7 | 96.8 | 97.2 | 97.5 | 97.7 | 98.0 |
