# Supplementary material for: Epidemiology of Trichomoniasis in South Korea and Increasing Trend in Incidence, Health Insurance Review and Assessment 2009-2014
Source: PLoS One. 2016 Dec 9;11(12):e0167938. doi: 10.1371/journal.pone.0167938 (PMC5148063; doi:10.1371/journal.pone.0167938)
Supplement: S2 Table — (DOC) [file pone.0167938.s004.doc]

| **Age**  **Group**  **(in**  **Years)** | **2009** | | | **2010** | | | **2011** | | | | **2012** | | | | | **2013** | | | | **2014** | | | | **2015** | | **Average incidence rate (per/100,000 persons)** | | |
| --- | --- | --- | --- | --- | --- | --- | --- | --- | --- | --- | --- | --- | --- | --- | --- | --- | --- | --- | --- | --- | --- | --- | --- | --- | --- | --- | --- | --- |
|  | **Total** | **Male** | **Female** | **Total** | **Male** | **Female** | **Total** | **Male** | **Female** | **Total** | | **Male** | **Female** | **Total** | **Male** | | **Female** | **Total** | **Male** | | **Female** | **Total** | **Male** | | **Female** | **Total** | **Male** | **Female** |
| Total | 260.4 | 23.7 | 501.0 | 264.3 | 22.5 | 510.0 | 255.0 | 19.6 | 493.9 | 249.4 | | 20.0 | 481.8 | 312.6 | 17.7 | | 611.0 | 319.1 | 15.7 | | 625.8 | 291.3 | 16.5 | | 569.0 | 278.9 | 19.4 | 541.8 |
| <20 | 20.2 | 0.5 | 41.9 | 22.2 | 0.5 | 46.0 | 22.0 | 0.5 | 45.7 | 23.0 | | 0.7 | 47.3 | 27.5 | 0.6 | | 56.7 | 27.9 | 0.7 | | 57.4 | 23.9 | 0.8 | | 48.8 | 23.8 | 0.6 | 49.1 |
| 20-29 | 367.3 | 13.9 | 747.6 | 376.2 | 14.4 | 768.4 | 379.9 | 12.3 | 780.7 | 377.9 | | 14.4 | 777.3 | 479.0 | 11.7 | | 997.2 | 477.9 | 12.0 | | 999.8 | 416.7 | 15.5 | | 868.5 | 410.7 | 13.4 | 848.5 |
| 30-39 | 414.4 | 30.2 | 820.7 | 423.0 | 27.9 | 840.8 | 415.0 | 24.8 | 827.4 | 402.6 | | 27.5 | 796.9 | 528.1 | 25.8 | | 1,058.4 | 558.9 | 25.2 | | 1,123.8 | 517.0 | 27.0 | | 1,038.0 | 465.6 | 26.9 | 929.4 |
| 40-49 | 474.8 | 51.4 | 916.5 | 472.2 | 46.7 | 917.5 | 434.8 | 38.5 | 850.6 | 414.3 | | 36.2 | 809.8 | 501.1 | 32.1 | | 989.2 | 507.3 | 27.6 | | 1,006.6 | 463.0 | 26.4 | | 917.3 | 466.8 | 37.0 | 915.4 |
| 50-59 | 286.1 | 44.1 | 528.8 | 297.3 | 41.9 | 552.6 | 286.4 | 36.2 | 536.3 | 285.1 | | 35.8 | 534.9 | 348.0 | 29.9 | | 667.6 | 349.3 | 25.2 | | 674.7 | 328.1 | 24.1 | | 633.6 | 311.5 | 33.9 | 589.8 |
| 60-69 | 93.7 | 13.5 | 167.7 | 97.6 | 14.8 | 174.5 | 95.5 | 14.3 | 171.3 | 106.4 | | 13.8 | 193.2 | 135.1 | 12.6 | | 250.6 | 140.6 | 8.8 | | 265.7 | 139.1 | 11.8 | | 259.7 | 115.4 | 12.8 | 211.8 |
| 70≤ | 30.8 | 5.0 | 47.2 | 32.6 | 4.3 | 50.7 | 33.5 | 3.8 | 52.7 | 37.7 | | 4.5 | 59.5 | 51.3 | 3.9 | | 82.8 | 56.6 | 2.6 | | 92.7 | 54.2 | 3.7 | | 88.2 | 42.4 | 4.0 | 67.7 |

**S2. Table. Number of patients with trichomoniasis per 100,000 person-years in HIRA, South Korea 2009-2015**
